# Biophysical regulation of *Chlamydia pneumoniae*-infected monocyte recruitment to atherosclerotic foci

**DOI:** 10.1038/srep19058

**Published:** 2016-01-20

**Authors:** Shankar J. Evani, Anand K. Ramasubramanian

**Affiliations:** 1 Department of Biomedical Engineering, University of Texas at San Antonio, San Antonio, TX 78249, USA.

## Abstract

*Chlamydia pneumoniae* infection is implicated in atherosclerosis although the contributory mechanisms are poorly understood. We hypothesize that *C. pneumoniae* infection favors the recruitment of monocytes to atherosclerotic foci by altering monocyte biophysics. Primary, fresh human monocytes were infected with *C. pneumoniae* for 8 h, and the interactions between monocytes and E-selectin or aortic endothelium under flow were characterized by video microscopy and image analysis. The distribution of membrane lipid rafts and adhesion receptors were analyzed by imaging flow cytometry. Infected cells rolled on E-selectin and endothelial surfaces, and this rolling was slower, steady and uniform compared to uninfected cells. Infection decreases cholesterol levels, increases membrane fluidity, disrupts lipid rafts, and redistributes CD44, which is the primary mediator of rolling interactions. Together, these changes translate to higher firm adhesion of infected monocytes on endothelium, which is enhanced in the presence of LDL. Uninfected monocytes treated with LDL or left untreated were used as baseline control. Our results demonstrate that the membrane biophysical changes due to infection and hyperlipidemia are one of the key mechanisms by which *C. pneumoniae* can exacerbate atherosclerotic pathology. These findings provide a framework to characterize the role of ‘infectious burden’ in the development and progression of atherosclerosis.

In addition to well-documented genetic and environmental factors, there is compelling evidence that, either directly or indirectly, microbial infections (‘infectious burden’) play an important role in the development and progression of atherosclerosis^1^. Infectious pathogens may contribute to atherosclerosis either by direct or indirect involvement: *C. pneumoniae* and human cytomegalovirus act directly on the arterial wall leading to endothelial dysfunction and foam cell formation, while these and others organisms such as *Helicobacter pylori* and influenza virus act through indirect mechanisms by inducing chronic systemic inflammation or by initiating an immune response against pathogenic antigens which share molecular patterns similar to human antigens^2^. Specifically, multiple lines of investigation implicate that *Chlamydia pneumoniae* infection is a highly likely risk factor for atherosclerosis including several *in vitro* cell culture^3^, seroepidemiological^4^, histopathological^5^, animal models of disease development and treatment^6^, and limited clinical intervention trials^7^. Despite such extensive correlatory evidence, the role of *C. pneumoniae* infection in atherosclerosis is poorly understood. Further, the clinical trials aimed at reversing atherosclerosis in patients with stable angina by antibiotic treatment failed leaving the results open to interpretation as either limited aetiologic role of pathogens, lack of antibiotic susceptibility or, most likely, late stage antibiotic treatment will not resolve an existing inflammatory condition^8^. Hence, it is imperative to elucidate the possible role microbes, which are found in close association to humans, as one of the key untested proatherogenic mechanisms^9^,^10^.

*C. pneumoniae* is an obligate intracellular bacterium which needs a host cell for survival, dissemination and further propagation. Following an initial infection, the infectious elementary bodies (EB) enter the host cell wherein they differentiate into non-infectious replicating reticulate bodies (RB) in the initial 4–8 hours. The RB multiply in an inclusion formed by utilizing host cell and bacterial machinery after 36–40 hours of infection and subsequently differentiates back to EB, before host cell dies to release the matured EBs. The EBs later infect other susceptible host cells at around 72 hours post infection^11^. Since *C. pneumoniae* is ubiquitous and reinfections of the lung are common, the infection draws repeated surges of immune cells into the lung^12^. *C. pneumoniae* is disseminated from the lungs to the vasculature through infected peripheral blood mononuclear cells to reach atherosclerotic foci^13^,^14^. *In vitro*, the infection cycle in monocytes/macrophages typically last up to 3 days during which the bacterium triggers the upregulation of a number of genes linked to the development of atherosclerosis, secretion of a plethora of inflammatory cytokines, and increase the expression of endothelial adhesion molecules^15^,^16^. In addition, infection also alters cholesterol homeostasis^17^, activation of LDL receptor^18^, and induce atherosclerosis in apo-E KO mice^19^. Taken together, these observations suggest that the changes in the physiology of the host cell due to *C. pneumoniae* infection may contribute to the development of atherosclerosis.

In this work, we test the hypothesis that the biophysical changes due to infection alter the interaction of monocytes with the endothelium, which is the first step in atherosclerosis. To this end, we characterize the effect of infection under hyperlipidemic conditions on the mechanics of rolling/adhesion of monocytes at physiologically relevant flow rates. We delineate the role of adhesion receptors and their distribution on the mechanics of monocyte-endothelial interactions, and propose a novel biophysical mechanism by which *C. pneumoniae* infection may promote atherogenic processes.

## Results

### Adhesion of *C. pneumoniae*-infected monocytes

**Figure 1:**
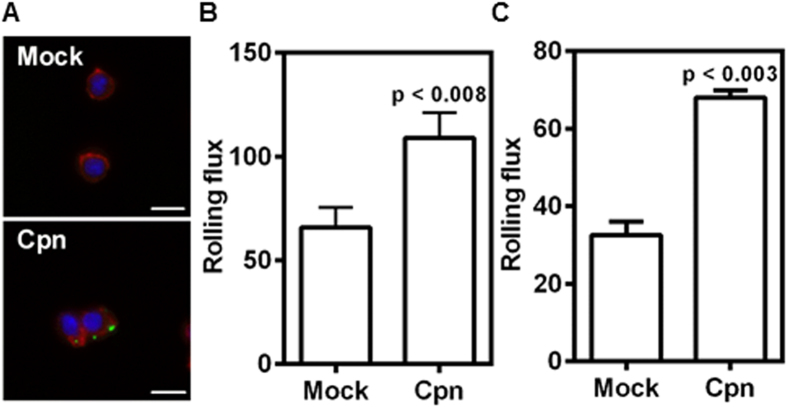
*C. pneumoniae* infection increases monocyte recruitment to E-selectin and endothelium under flow. Monocytes were infected with mock PBS or Chlamydial EB (MOI 1) for 8 h. **(A)** The cells were stained for *Chlamydia pneumoniae* (green), actin (red), and nucleus (blue). Representative image of Mock and Cpn infected cells are shown (Scale bar, 10 μm). **(B,C)** In another experiment the cells were re-suspended at a concentration of 0.5 million/ml in media and perfused at 1 dyn/cm^2^ over micro-channels coated with E-selectin **(B)**; and over confluent aortic endothelium activated for 4 h with 20 ng/ml of TNFα **(C)**. The number of cells rolling on the surface was obtained from video microscopy (20 fps) for 5 minutes. The results are mean ± SEM of one representative experiment performed in triplicate, and the experiments were repeated five times. The statistical significance in the parameters between the groups was shown as p value from t test.

As an intracellular pathogen, *C. pneumoniae* forms inclusions in the cytoplasm of the infected cells. We observed that an MOI 1 was sufficient to see bacteria in >90% of cells, and within 8 h of infection, chlamydia was visible (Fig. 1A)^20^. It has previously been shown that *C. pneumoniae* infection increases the adhesion of monocytes to endothelium under static conditions, though it is now well established that the mechanics of adhesion under physiological flow conditions can be very different^21^. We evaluated the effect of *C. pneumoniae* infection under flow on the interaction monocytes with endothelial cells and also the major endothelial adhesion receptor, E-selectin. All experiments were performed on monocytes infected for 8 h during which infection is established and a strong proinflammatory response is observed *in vitro*^20^. We perfused monocytes at a shear stress of 1 dyn/cm^2^ for 5 min over E-selectin and TNFα-activated aortic endothelium, and assessed the number of rolling cells per unit time. We observed a 2-fold increase in the number of infected monocytes rolling on these surfaces compared to uninfected cells (Fig. 1B,C).

### Infected monocytes roll uniformly on E-selectin and endothelium

**Figure 2:**
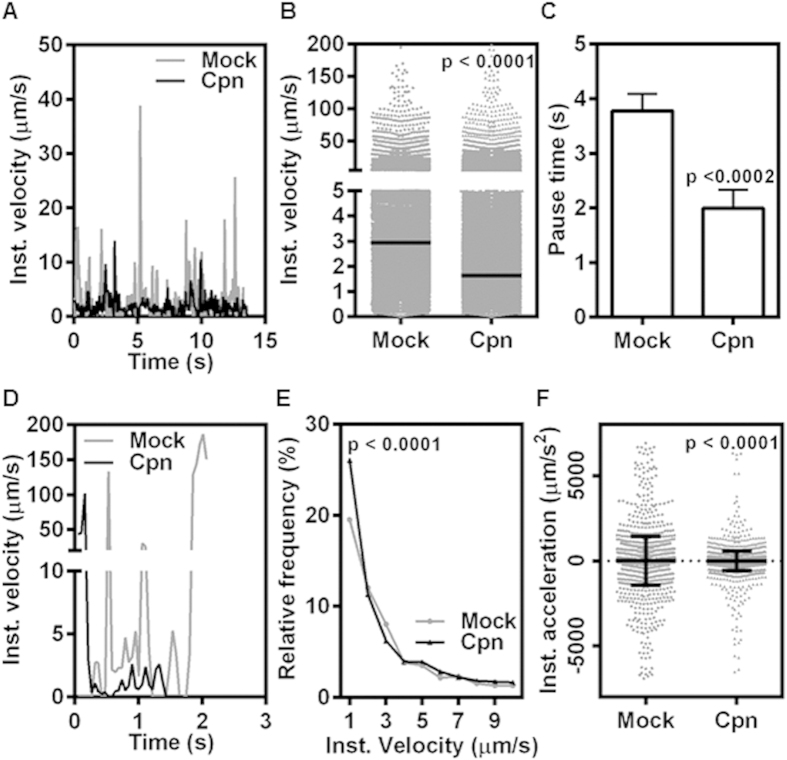
*C. pneumoniae*-infected monocytes display unsteady rolling on E-selectin. Uninfected (mock) or infected (Cpn) monocytes were perfused at 1 dyn/cm^2^ on micro-channels coated with E-selectin **(A–C)** and micro-channels with activated confluent aortic endothelium **(D–F)**. The cellular interactions were recorded at 20 fps for 5 minutes. Representative rolling velocity profile **(A)**; distribution of instantaneous velocities of monocyte rolling with median **(B)**; and pause time with mean ± SEM **(C)** on E-selectin. Representative rolling velocity profile **(D)**; instantaneous velocity frequency distribution **(E)**; and instantaneous acceleration **(F)** on activated endothelium. The results are mean ± SD of one representative experiment (unless mentioned otherwise) performed in triplicate, and the experiments were repeated five times. The statistical significance in the parameters between the groups was shown as p value from t test.

To understand the mechanistic basis for increased rolling of infected monocytes, we performed a frame-by-frame analysis of cells rolling on E-selectin-coated surfaces. E-selectin on endothelial surface is a major receptor for infiltrating immune cells including monocytes^22^,^23^. We studied the kinetics of rolling based on instantaneous velocities obtained by following the displacements of uninfected and infected monocytes as they roll on E-selectin surface. Fig. 2A shows representative profile of instantaneous velocity at a shear stress of 1 dyn/cm^2^. We observe that the uninfected (Mock) cells show predominantly saltatory motion followed by short pauses while infected (Cpn) cells rolled continuously and smoothly. The instantaneous velocities obtained from hundreds of such tracks were found to be distributed over a broad range (0.1–100 μm/s). This distribution revealed that significantly more infected cells rolled at a lower velocity than uninfected cells, and the median rolling velocity of the infected cells was 2-fold lower than that of the uninfected cells (Fig. 2B). However, between rolling events, infected cells paused for shorter time than uninfected cells (Fig. 2C). Thus, lower rolling velocity and shorter pauses mean that the infected cells roll more smoothly on E-selectin surface.

Next, we used TNF-α-activated aortic endothelium to simulate the adhesive phenotype of atherosclerotic foci to characterize the recruitment of *C. pneumoniae*-infected cells. We perfused uninfected or infected monocytes on activated endothelium, and analyzed the interactions as described above. We observed that, similar to that on the E-selectin-coated surface, infected-monocytes displayed more uniform rolling and lower rolling velocities than uninfected monocytes (Fig. 2D,E). The uniformity in rolling interactions of infected cells is best quantified by instantaneous acceleration, which is a direct measure of start/stop phenomenon. As can be seen in Fig. 2F, infected cells have a narrower and smaller range of acceleration than uninfected cells, i.e., they roll much more smoothly and uniformly on endothelium.

### *C. pneumoniae* infection alters lipid raft distribution

**Figure 3:**
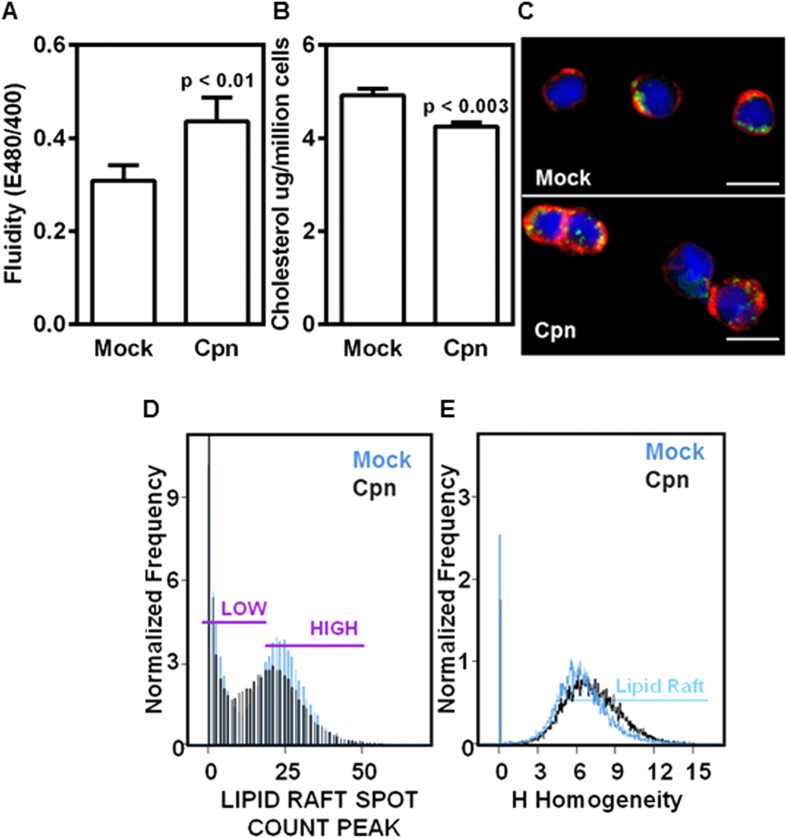
*C. pneumoniae* infection increases membrane fluidity and uniformity of lipid raft distribution. Uninfected (mock) or infected (Cpn) monocytes were analyzed for fluidity of membrane using a fluorescence plate reader and the results were plotted as ratio of emissions at 480 and 400 nm **(A)**; analyzed for total cholesterol using fluorescence plate reader **(B)**; stained for membrane lipid rafts (green), actin (red), and nucleus (blue), and visualized by confocal microscopy (Scale bar, 10 μm) **(C)**. The results are mean ± SEM of five different experiments performed in triplicate. The statistical significance in the parameters between the groups was shown as p value from t test. In another experiment monocytes were stained for lipid rafts and analyzed for lipid raft distribution by spot count analysis **(D)**; and homogeneity **(E)** by imaging flow cytometry. The results are from one representative experiment performed in triplicate, and all the experiments were repeated five times.

Since membrane properties play a direct role in cell adhesion, we assessed the effect of infection on membrane fluidity. As shown in Fig. 3A, the fluidity of the membrane of infected cells increased by more than 40% compared to that of the uninfected cells. We also observed that the increase in fluidity in infected cells is attributable to a decrease in cholesterol, possibly due to cholesterol efflux observed during the chlamydial infection cycle^24^ (Fig. 3B).

These changes in cholesterol content and membrane fluidity lead us to estimate the lipid raft distribution, which essentially are the floating islands rich in cholesterol and where major adhesion receptors often are concentrated. The lipid rafts were identified using cholera toxin B against ganglioside GM1, which is known to be specifically bound in the rafts. We observed that lipid rafts are concentrated at focal points in uninfected cells but gets dispersed during infection (Fig. 3C). The quantification of the distribution of individual rafts showed that the individual rafts become smaller in size, and they get more evenly distributed throughout the surface of infected cell membranes (Fig. 3D,E). These results show that *C. pneumoniae* infection in monocytes affects the biophysical properties of the membrane with changes in fluidity and lipid raft distribution.

### CD44 distributed in lipid rafts predominantly mediates monocyte rolling

**Figure 4:**
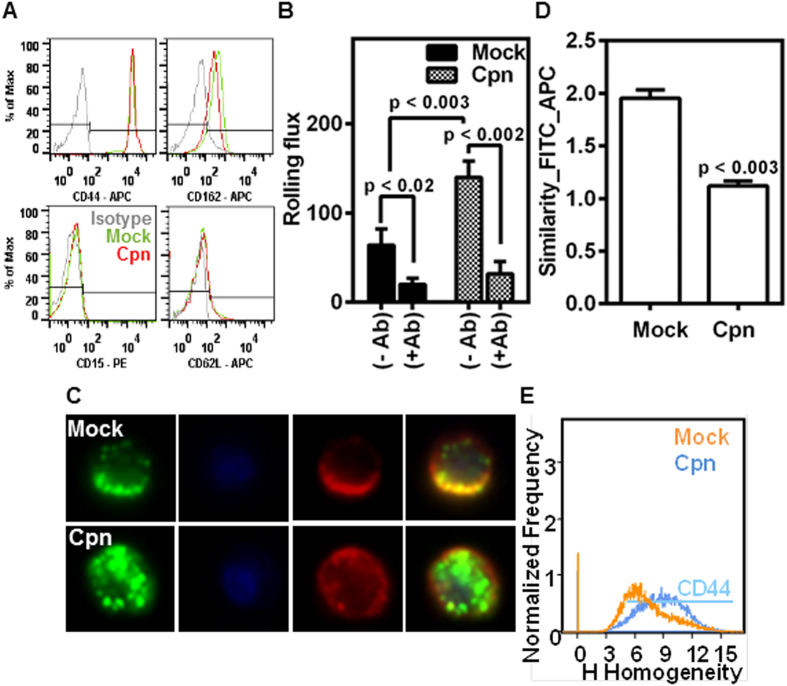
CD44 mediates rolling and is more uniformly distributed in infected cells. Uninfected (mock) or infected (Cpn) monocytes were stained with CD44, CD15, CD162, or CD62L antibodies, and analyzed by flow cytometry **(A)**. In another experiment, the cells (mock or Cpn) after 8 h of infection were blocked with 0 or 1 μg of CD44 antibody per million cells for 30 minutes, washed and re-suspended at a concentration of 0.5 million/ml in media and perfused on activated endothelium at 1 dyn/cm^2^, and the rolling interactions were quantified by video microscopy **(B)**. **(C–E)** Cells after 8 h of infection were stained for CD44 (red), lipid raft (green) and nucleus (blue) and visualized by imaging flow cytometry, with representative images **(C)**; co-localization of CD44 and lipid rafts **(D);** and homogeneity in the distribution of CD44 **(E)** are shown. The results are mean ± SEM **(B,D)** of one representative experiment performed in triplicate, and all the experiments were repeated three times. The statistical significance in the parameters between the groups was shown as p value from ANOVA **(B)** or t test **(D)**.

Next, we evaluated the effect of infection on the expression levels and the surface distribution of monocyte receptors that mediated endothelial and E-selectin adhesion. Using flow cytometry, we quantified the expression levels of various co-receptors of E-selectin, namely CD44, CD162, CD62L and CD15. We observed that CD44 levels were 100-fold higher than other receptors in both uninfected and infected monocytes, suggesting that CD44 may be predominantly involved in the interaction of monocytes with E-selectin or endothelium (Fig. 4A) (Fig. S1). We also observed that infection did not appreciably alter the expression levels of CD15, CD62L and CD162 from the already low levels and increased CD44 expression levels only modestly (~10%) (Fig. 4A) (Fig. S1). To further confirm the dominance of CD44 on rolling interactions, we perfused uninfected/infected monocytes treated with CD44 antibody; and observed that such blocking nearly abolished the rolling of monocytes under flow (Fig. 4B).

Having established that CD44 is a key mediator of monocyte rolling on endothelium, we sought to determine the effect of infection on CD44 distribution on the cell membrane. We stained both CD44 receptor and lipid rafts, and simultaneously visualized the localization of CD44 in lipid rafts. Consistent with previous reports, we observed that CD44 was concentrated in the lipid rafts (Fig. 4C,D)^25^. With infection, as lipid rafts and CD44 get more evenly distributed (Fig. 3D), CD44 also disengages from the lipid rafts as seen from the decrease in the overlap of intensities of CD44 and lipid rafts from the exact spatial location on the membrane (Fig. 4D). Consequently, the even redistribution of CD44 is quantified by a substantial increase in the homogeneity index (Fig. 4E). Interestingly, only CD162 is concentrated in the rafts, although lipid raft redistribution due to infection had no effect on CD162, CD15 or CD62L (Fig. S2).

### LDL increases membrane fluidity and CD44 homogeneity in infected, but not uninfected, cells

**Figure 5:**
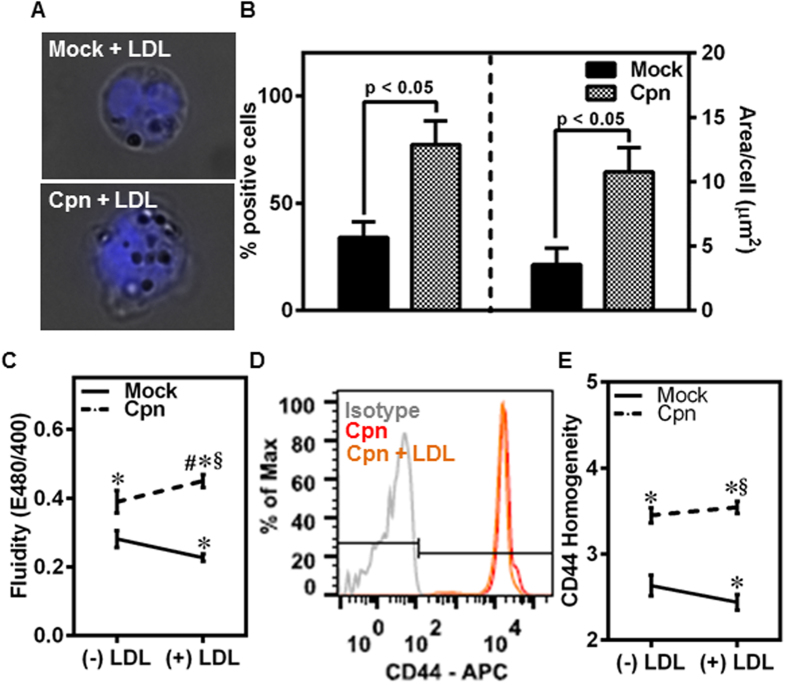
LDL increases CD44 homogeneity in infected, but not uninfected, monocytes. **(A–E)** Monocytes were infected with mock PBS or Chlamydial EB (MOI 1) for 4 h and further incubated for 4 h with 0 or 100 μg/ml of LDL. The cells were stained for: LDL with Oil red O (droplets) and nucleus (blue) **(A)**; quantitative uptake of LDL using ImagePro software **(B)**; membrane fluidity estimation using fluorescent plate reader **(C)**; CD44 expression assayed by flow cytometry **(D)**; and CD44 distribution on the membrane by imaging flow cytometry **(E)**. The results are mean ± SEM **(B,C,E)** of three different experiments performed in triplicate. The statistical significance in the parameters between the groups was shown as p value from t test **(B)**. The *, # and § denote statistically significant change in the parameters of the groups in comparison to mock, Cpn and mock + LDL respectively (p < 0.05, ANOVA) **(C,E)**.

Experimental and clinical studies indicate that *C. pneumoniae* infection exacerbates atherosclerosis under hyperlipidemic conditions^7^. To test the effect of hyperlipidemia together with infection on monocyte adhesion, first we incubated *C. pneumoniae*-infected monocytes with 100 μg/ml of LDL and analyzed the uptake by Oil-red-O staining. We observed that although both uninfected and infected monocytes take up LDL, infection significantly increases the number of cells containing LDL and also the amount of LDL in each cell (Fig. 5A,B). Further, LDL did not affect the infectivity of *C. pneumoniae* in monocytes (Fig. S3).

Next, we estimated the effect of LDL uptake on changes in the fluidity of the membrane, lipid raft distribution and CD44 homogeneity. As expected, in the uninfected cells, LDL uptake decreased the fluidity of the membrane due to the sequestration of cholesterol from added LDL into the membrane (Fig. 5C). Interestingly, in the infected cells, LDL uptake increased the fluidity of the membrane even more than that due to infection alone. This may possibly be because LDL exposure further enhances the sequestration of membrane cholesterol by the chlamydial inclusions^26^,^27^. Thus, the fluidity of the membrane in infected cells exposed to LDL was 2-fold higher than that of uninfected cells. When we analyzed the expression and distribution of CD44 on the membrane, we observed that the total expression levels are unaffected by exposure to LDL (Fig. 5D). Expectedly, since CD44 is concentrated on the lipid rafts, the distribution of CD44 follows the same trend as the fluidity, i.e., CD44 is distributed more homogeneously on the surface of infected cells than on uninfected cells though (Fig. 5E). Together, *C. pneumoniae* infection and LDL exposure additively enhance membrane fluidity and CD44 homogeneity.

### LDL increases infected monocyte adhesion to endothelium

**Figure 6:**
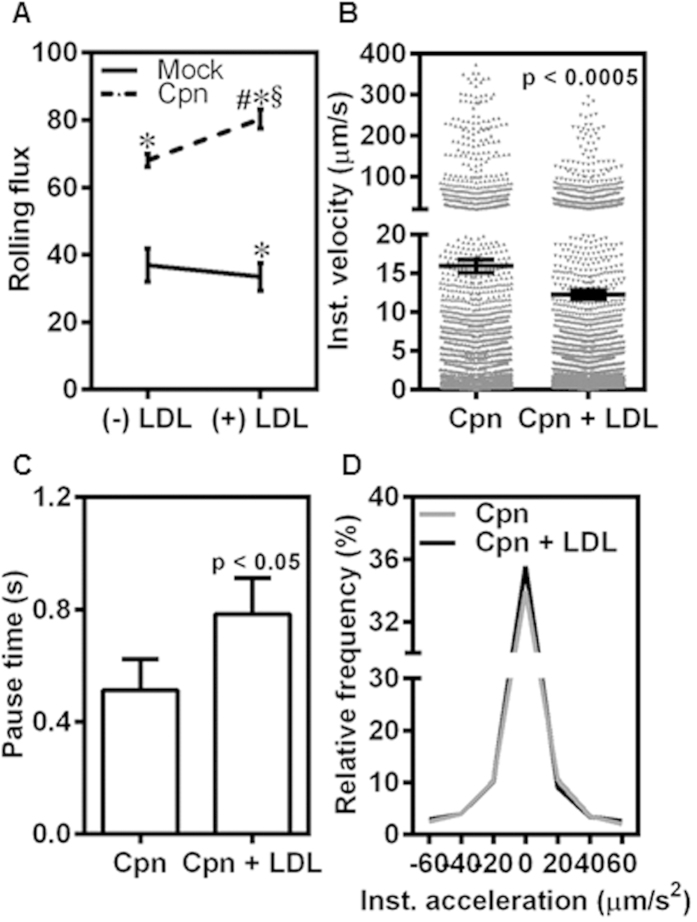
LDL increases uniform rolling of infected monocytes on endothelium. **(A)** Monocytes were infected with mock PBS or Chlamydial EB (MOI 1) for 4 h and further incubated for 4 h with 0 or 100 μg/ml of LDL. The cells were then re-suspended at a concentration of 0.5 million/ml in media and perfused on activated aortic endothelium at 1 dyn/cm^2^, and the interactions were visualized by video microscopy. Rolling flux of uninfected (Mock) and infected (Cpn) conditions with or without LDL. The *, # and § denote statistically significant change in the parameters of the groups in comparison to mock, Cpn and mock + LDL respectively (p < 0.05, ANOVA). **(B-D)** Instantaneous velocity **(B)**; pause time **(C)**; and acceleration **(D)** of infected (Cpn) and infected with LDL treatment (Cpn + LDL) conditions are shown. The statistical significance in the parameters between the groups was shown as p value from t test **(B,C)**. The results are either mean ± SEM **(A-C)** or relative frequency **(D)** of one representative experiment performed in triplicate, and all the experiments were repeated three times.

To assess the consequences of LDL uptake on *C. pneumoniae*-infected monocyte recruitment to the endothelium, we characterized the rolling and adhesion on to TNFα-activated endothelium at 1 dyn/cm^2^. We observed that LDL exposure did not have any effect on the number of uninfected cells rolling on the endothelium although it significantly increased due to infection possibly because LDL is taken up largely by infected cells (Fig. 6A). We also observed that infected-monocytes incubated with LDL rolled more slowly, and paused longer when compared to infected cells without exposure to LDL (Fig. 6B,C). Together, this resulted in a more uniform rolling of infected, LDL-treated cells as demonstrated by smaller instantaneous acceleration (Fig. 6D). Together with data from Figs 5 and 6, these results suggest that increase in fluidity of the membrane and a more homogeneous distribution of CD44 may render infected cells exposed to LDL roll more slowly and uniformly on the endothelium.

**Figure 7:**
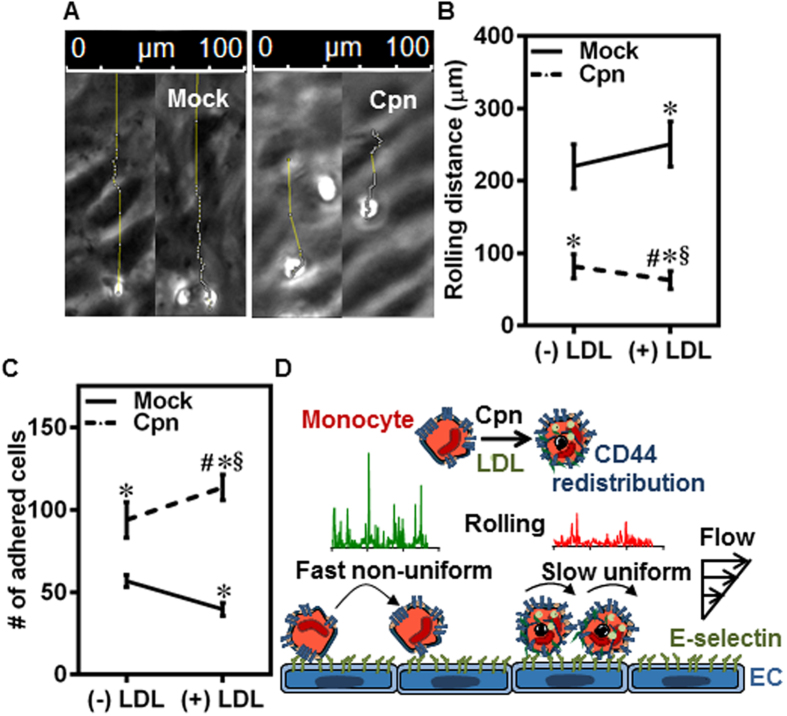
LDL increases monocyte rolling and adhesion to endothelium. **(A–C)** Monocytes were infected with mock PBS or Chlamydial EB (MOI 1) for 4 h and further incubated for 4 h with 0 or 100 μg/ml of LDL. The cells were then re-suspended at a concentration of 0.5 million/ml in media and perfused on activated aortic endothelium at 1 dyn/cm^2^, and the interactions were visualized by video microscopy. Micrograph of tracks for uninfected (Mock) and infected (Cpn) conditions **(A)**; distance rolled by the cells before adhering firmly **(B)**; and number of cells firmly adherent on endothelium under various conditions **(C)**. The results are mean ± SEM of three different experiments performed in triplicate. The *, # and § denote statistically significant change in the parameters of the groups in comparison to mock, Cpn and mock + LDL respectively (p < 0.05, ANOVA). **(D)** Proposed biophysical mechanism for the increased adhesion of infected monocytes to the endothelium in the presence of LDL.

On endothelium, rolling translates to firm adhesion/arrest as seen from the individual tracks of rolling cells on the endothelium (Fig. 7A). We calculated rolling distance as the distance traveled by the monocytes before coming to full arrest, which represents the engagement of adhesion receptors on the monocytes with endothelium (Fig. 7B and Fig. S4). As shown in Fig. 7B, LDL treatment had opposite effects on the rolling distance in uninfected and infected monocytes. While LDL uptake in uninfected cells disfavored the conversion of rolling to firm adhesion, LDL uptake in infected cells favored the transition from rolling to firm adhesion. As a consequence, more infected cells exposed to LDL were seen firmly adhered to the endothelium than in the absence of infection or without LDL (Fig. 7C). Thus, *C. pneumoniae* infection increases both recruitment/rolling of monocytes and eventual firm adhesion on to the endothelium by 3-fold, under hyperlipidemic conditions. These results confirm that the primary reason for increased adhesion of monocytes to the endothelium is the redistribution of CD44. Together, we propose that purely biophysical transformations due to *C. pneumoniae* infection under hyperlipidemic conditions may increase the recruitment of infected monocytes to activated endothelium (Fig. 7D).

## Discussion

*Chlamydia pneumoniae* is an intracellular respiratory pathogen implicated in atherosclerosis. *C. pneumoniae* first infects lung epithelium, followed by neutrophils and resident macrophages, and is carried into the circulation by peripheral blood monocytes before getting lodged in the atherosclerotic foci^28^. Amongst other evidences, both viable bacteria and bacterial DNA have been isolated from atheromatous arteries suggesting a possible role of *C. pneumoniae* in atherosclerosis^29^. In this work, we have demonstrated that *C. pneumoniae* infection, particularly under hyperlipidemic conditions, favors endothelial adhesion of monocytes because of dynamic changes in membrane architecture.

Tethering and rolling of circulating monocytes on to the activated endothelial surface are the first steps in the multistep cascade of adhesion and signaling events, and are prerequisites for eventual adhesion and transmigration^30^. The transient rolling interactions of leukocytes are mediated by a gamut of receptors which demonstrate certain redundancy including CD44, CD62L (L-selectin), CD162 (PSGL-1), and CD15 on leukocyte surface that bind to selectins on activated endothelial surface. We found that CD44 is the major receptor expressed by monocytes for endothelial E-selectin; as others are expressed at much lower levels, and the interaction under flow is significantly low in the absence of CD44. Consistent with previous reports showing E-selectin as a major endothelial receptor for leukocyte rolling, we observed aortic endothelial cells predominantly expressing this molecule when activated with TNF-α (Data not shown). The binding of CD44 with E-selectin contributes significantly to the rolling, arrest, and migration of inflammatory T-lymphocytes and neutrophils^31^,^32^,^33^,^34^. Recently, it was shown that, just as with selectins, shear stress stabilizes the high affinity conformation of CD44, which is essential for cell rolling on endothelium^35^.

Rolling is an inherently unstable state characterized by jerky, stop and go motion of short timescales^36^. Cells roll as bonds in the rear break due to the force and torque generated by hydrodynamic flow, and these bonds are replaced by new adhesive bonds that form in the front. The stark differences in the peaks and valleys of the rolling velocity between uninfected and infected cells on E-selectin or endothelium displayed in Fig. 2 suggest that the biophysical mechanisms that govern the mechanics of CD44-selectin bond formation and breakage, namely cellular deformation and binding kinetics, are significantly affected by *C. pneumoniae* infection^37^.

Cells are viscoelastic, and both membrane and cytoplasm contribute to cellular mechanics. Under shear flow conditions, cells attached to a surface deform. The elastic deformation increases the cell-surface contact area, and the viscous component produces a delayed response to applied force. Hence, more viscous or compliant cells roll slower and smoother in comparison to stiffer cells or rigid beads of the same receptor density^38^,^39^. As shown in Fig. 3, infected cells have lower cholesterol content and increased membrane fluidity, which is associated with a decrease in Young’s modulus or cell stiffness^40^. Further, when *C. pneumoniae* forms inclusions, it ‘hijacks’ the host cytoskeleton leading to a less organized internal structure or a more viscous cytoplasm^41^. Thus, we infer that changes in cellular viscoelasticity due to infection may flatten the cells, create a larger footprint which translates to more smooth and slow rolling compared to uninfected cells.

The binding kinetics depends on the density and distribution of the receptors on the cell surface. Since the modest increase in the density of CD44 alone (~10%, Fig. S1) could not account for the large (2-fold) changes in the rolling parameters (Fig. 2) of infected cells; we investigated the changes in the distribution of CD44 on cell surface due to infection. Consistent with previous studies, we observed that CD44 is co-localized with lipid raft microdomains^25^. Lipid rafts are fluctuating assemblies of sphingolipid, cholesterol, and proteins that serve as a platform to host receptor clusters involved in adhesion and signaling^42^. The jerky motion observed during rolling is a signature of formation, loading and breakage of individual bond clusters^43^. It has been shown by experiments and by simulations that a fewer bond clusters mean that as all but one becomes load bearing, it causes sudden slowing down or stoppage; and conversely, the breakage of the solitary load bearing cluster result in sudden acceleration or jump^43^,^44^. In light of these observations, we found that the non-uniform or jerky motion in uninfected cells correlated well with the heterogeneous distribution of lipid raft-associated CD44 on the cell surface. Conversely, the more stable and steady rolling observed in infected cells correlates well with the redistribution of lipid rafts and increased homogeneity of CD44 (Figs 3 and 4). Thus, it may be deduced that, more uniform force loading on infected cells may also contribute to more smooth rolling compared to uninfected cells.

Of clinical relevance, hyperlipidemia and *C. pneumoniae* infection are considered as co-risk factors for atherogenesis based on epidemiological data and *in vivo* models^45^. Our data (Fig. 5) shows that under hyperlipidemic conditions simulated with LDL, *C. pneumoniae* infection nearly doubles membrane fluidity and CD44 homogeneity. It should be mentioned that the LDL concentration chosen in our study is 10–20 fold lower than that in plasma. Based on aforementioned implications of infection on cell and bond micromechanics, it may be argued that this change in CD44 redistribution in infected, LDL-exposed cells may account for the increase in the number of infected cells rolling on the endothelium by 3-fold compared to uninfected cells (Fig. 6). As a consequence of this shift to slower and steady rolling due to infection, 3-fold more infected cells adhered firmly on the endothelium (Fig. 7). Taken together, these results suggest that the biophysical changes which modulate rolling interactions are sufficient to increase the arrest and adhesion of infected monocytes under hyperlipidemic conditions, thus accelerating atherogenesis.

In summary, we have shown that *C. pneumoniae* infection alters monocyte recruitment to E-selectin/activated endothelium under physiologically relevant conditions. We demonstrate that the increased adhesion of infected monocytes is due to biophysical changes on the membrane that alter CD44 distribution on lipid rafts and the fluidity of membrane, which together alter the dynamics of CD44–E-selectin interactions. We also demonstrate that the uptake of LDL by infected cells propels the changes further in the same direction. Together, we conclude that *C. pneumoniae* infection, particularly under hyperlipidemic conditions, enhances monocyte adhesion to endothelium by altering the fluidity of the membrane, lipid raft-associated CD44 distribution, and the ensuing transient rolling interactions, which is the first step in atherogenesis. Our data provide a heretofore unknown biophysical basis for a direct mechanism by which *C. pneumoniae*-infection can exacerbate atherosclerosis. In a larger context, our results may provide a framework for etiologic understanding of infectious agents as a causative agent for atherosclerosis.

## Materials and Methods

### Cells

Human blood samples, without any donor identifiers, were obtained after their informed consent, and the buffy coat was isolated by the South Texas Blood and Tissue Bank (STBT), San Antonio, TX. Buffy coat was procured from STBT. All the methodologies and procedures were approved by Institutional Review Board (IRB), The University of Texas at San Antonio, San Antonio, TX (protocol #12-227), and the experiments were conducted in accordance with these appropriate guidelines. Monocyte negative isolation kit ll from Miltenyi was used to isolate monocytes from buffy coat. RPMI 1640 (ATCC) media supplemented with 10% HI FBS (Life Technologies) was used to culture monocytes. Briefly, buffy coat from anonymous healthy donors was obtained. Buffy coat bags had (~45 ml) leukocytes with red blood cells and platelets. To isolate peripheral blood mononuclear cells (PBMCs), buffy coat was diluted to a final volume of 140 ml with Dulbecco’s phosphate buffered saline (D-PBS without calcium and magnesium; Corning) with 2mM EDTA (Sigma) (PBS-EDTA). 15 ml of Ficoll-Paque Plus (GE Healthcare) was added to each of 4, 50 ml Leukosep tubes (Greiner Bio-One) and centrifuged at 1000g for 1 minute. 30 ml diluted buffy coat was added to these tubes. Tubes were then centrifuged at 1000g for 10 minutes at room temperature with the brake off. PBMCs were collected to four fresh 50 ml centrifuge tubes and washed at 100g for two times with PBS-EDTA to remove contaminating platelets. Monocytes were isolated from PBMCs by negatively labelling other cells with antibodies, then tagging them with microbeads and proceeding to magnetic isolation on LS columns according to manufacturer’s instructions (Miltenyi Monocyte- negative isolation kit ll). The cell viability was estimated using trypan blue exclusion assay (Countess automated cell counter, Life Technologies, Grand Island, NY). For purity estimation, monocytes were washed twice in PBD-EDTA and stained with anti-human CD14 antibody with appropriate isotype control at recommended dilution (Miltenyi). These cells comprise almost CD14 + monocytes (>90%) as determined by flow cytometry (BD FACS Caliber) and Flow Jo software.

Primary human aortic endothelial cells (HAECs) were obtained along with media and supplements from Life Line cell Technologies and cells were further passaged and stored at −80 °C within 3 passages (8 doublings) until further use according to manufacturer’s instructions.

### Bacteria

*Chlamydia pneumoniae* TW183 EB was obtained from University of Washington (Seattle, WA), and aliquoted and stored at −80 °C. *C. pneumoniae* stock titer was determined following established protocols using TT401 antibody^21^,^46^.

### *C. pneumoniae* infection and LDL treatment of monocytes

Human Monocytes were infected (Cpn) in a ultralow attachment T25 flask (Corning) with 2 × 10^7^/flask and infected with 2 × 10^7^ inclusion forming units (IFU) of Chlamydial EB in a total volume of 600 μl/flask (Multiplicity of Infection, MOI 1). The plate was incubated for 2.5 hours at 37 °C in a humidified 5% CO_2_ incubator with intermittent shaking for every 15 minutes to ensure efficient infection. After 2.5 hours post infection, cells were washed and spun down at 300 x g for 10 min in centrifuge. The monocytes were then re-suspended in complete media at a concentration of 2 × 10^6 ^cells/ml and further incubated for 5.5 more hours in ultralow attachment T25 flask. Post incubation cells were processed for various experiments. Monocytes incubated without chlamydial EBs were used as controls (Mock).

For LDL treatment experiments, 0 or 100 μg of LDL (Sigma, catalog L7914) was added to each ml of cells at 4 hours post infection and placed on rocker at 37 °C and incubated for 4 more hours, before running any experiments.

Monocytes with or without LDL treatment after 8 hours post infection were fixed in 4% formaldehyde in PBS for 10 minutes. The cells were cytospun (Thermo scientific, USA) on microscope glass slides (Thermo scientific), washed and blocked in 2% BSA in PBS for 30 minutes. The slides were then incubated with 200 μL of mouse monoclonal anti-chlamydial antibody (TT401- University of Washington, Seattle) solution with 1:500 dilution of antibody for 2 h. After staining, the slides were washed two times with PBS-T (PBS with 0.5% Tween-20), and further incubated in AF-488 conjugated goat anti-mouse IgG-secondary antibody (Life technologies) at 1 μg in 100 μL Perm wash (BD Bio) for 1 hour. The slides were further stained for actin using AF-633 phalloidin (Life technologies) and nucleus with DAPI (Life technologies) at recommended dilutions for 30 minutes. The samples were then washed with PBS-T and mounted with Fluorsave (Millipore). Fluorescent microscopic images were capture using Zeiss 510-confocal microscope or Fluorescent microscope (Leica, USA).

### Perfusion assays

IBIDI Treat Vl^0.4^ slides (IBIDI) were coated with 1 μg/mL of rh E-selectin (R & D Biosystems, catalog 724-ES), for 2 hours at RT and blocked with 2% BSA (AMRESCO) in PBS-EDTA for another 1 hour. The cells cultured to confluence in IBIDI Treat Vl^0.4^ slides (IBIDI) were treated with 20 ng/mL of rh TNF-α (R & D Biosystems, catalog 210-TA), for 4 h at 37 °C. The flow chamber with IBIDI slide was assembled on top of an inverted microscope attached to a time-lapse digital camera (FX-360; Leica Microsystems). A suspension consisting 0.5 × 10^6^ monocytes/ml were then drawn through the perfusion chamber with E-selectin/endothelium using a syringe pump (Harvard Apparatus) at a constant flow rate corresponding to a wall shear stress of 1 dyn/cm^2^. Monocyte interactions with E-selectin/endothelium were captured by video recording at 20 fps using a bright-field microscope at 10 × magnification within first 5 minutes in 5 different fields of view (1 × 1 mm^2^). The videos/images were analyzed offline using Image Pro Analyzer. For rolling analysis, cutoff for a rolling event was established as a cell displaced for less than 8 microns (i.e., 1 cell diameter) in two frames. Rolling flux was determined as number of cells rolling in one minute in a 1.2 mm^2^ area. Instantaneous velocity and acceleration was calculated from the distance swept by cell between two consecutive frames. Pause time was calculated as duration for which the cells moved for less than one cell diameter during a rolling event. Rolling distance for cells rolling on endothelial cells was calculated as distance covered by cell before firmly adhering to the endothelium.

### Estimation of membrane fluidity

Pyrene decanoic acid- membrane fluidity assay kit (Marker gene technologies, catalog M0271) was used to probe the membrane fluidity of the cells. Approximately 2 × 10^5 ^cells were used for each experimental condition. The cells from above were spun down and 20 μM solution of the probe was used with 0.1% pluronic F127. The cells were resuspended in 300 μL complete media containing the probe. The cells were incubated at room temperature for 20 min in the dark. The cells were then washed twice with 500 μL complete media and the final cell suspension of 200 μL cells in PBS were put in a black well clear bottom 96 well plate (Corning). Fluorescence intensity measurements were made at 360 nm (excitation)/372 & 470 (emission: monomer & eximer) using a Synergy2 plate reader (BioTek). The ratio of monomer to eximer emission intensity were plotted to estimate the changes in membrane fluidity.

### Measurement of lipid raft distribution

Monocytes were spun down and adjusted to a final concentration of 10^6^/100 μL. After blocking in 2% BSA in PBS for 10 minutes, the cells were washed two times with PBS and stained with Alexafluor-488 lipid raft labeling kit (Life technologies, catalog V34403) following manufacturer’s instructions with two modifications: using twice the concentration of components and half the recommended volume of media volume. The cells were washed two times with PBS-EDTA, fixed with 4% formaldehyde and stained for nucleus with DAPI for 10 min and re-suspended in 500 μl PBS-EDTA. Fluorescent images of at least 25,000 cells were captured using Amnis flow cytometry and analyzed for lipid raft distribution using Image stream software.

In some experiments, the fixed cells were cytospun (Thermo scientific) on to microscope slides (Thermo scientific), washed and blocked in 2% BSA in PBS for 30 minutes. The slides were stained for actin using AF-633 Phalloidin at recommended dilution for 30 minutes. The samples were then washed with PBS-T and mounted with Fluorsave. Fluorescent microscopic images were capture using confocal microscope (510-Zeiss) or Fluorescent microscope (Leica).

### Measurement of surface receptor expression levels

Monocytes from 8 hours post infection with or without LDL or after lipid raft staining were stained for co-receptors for endothelial receptors. The cells were spun down and blocked in 2% BSA in PBS-EDTA for 10 minutes. The cells were then incubated at 4 °C with fluorescently tagged mouse monoclonal anti-human CD44 (clone DB105) or CD15 (clone VIMC56) or PSGL-1 (clone REA319) or CD62L (clone 145/15) antibodies (Miltenyi Biotec) with appropriate isotype controls (Miltenyi Biotec) at recommended dilution along with DAPI for 10 minutes. After staining, the cells were washed two times with PBS-EDTA, and suspended in 4% formaldehyde for 10 minutes. Post fixing, cells were spun down and resuspended in 500 μl PBS. Fluorescent images of at least 25,000 cells were captured using Amnis flow cytometry and the data was analyzed using Image stream software.

### Measurement of cellular cholesterol content

Monocytes were spun down and adjusted to a final concentration of 10^6^/100 μL. The contents of intracellular total cholesterol (TC) and free cholesterol (FC) were detected by enzyme-fluorescence method using Amplex Red Cholesterol assay kit (Molecular Probes, catalog A12216) as described by the manufacturer, with samples in a black well clear bottom 96 well plate and reading were taken using a fluorescent micro-plate reader. The cholesterol contents of cell were determined from the standard curve obtained using 1–10 μg/ml cholesterol standard.

### Measurement of monocyte LDL uptake

Monocytes were spun down and adjusted to a final concentration of 10^4^/100 μL, fixed with 4% formaldehyde and cytospun onto microscopic slides. The cells were then incubated with oil red O stain (Life Line Cell Technologies, catalog CM-0054) and DAPI for 10 minutes and washed two times with PBS. The slides were then mounted with FluorSave and images from at least 5 different locations were taken using fluorescent microscope and were analyzed using ImagePro software to manually estimate percentage cells with LDL and quantify LDL uptake by measuring the area of LDL per cell for at least 60 cells per condition.

### Statistical analysis

All the experiments were done in triplicates with minimum of three donors and up to five donors on different days. Measurements from two groups were compared with a paired Student *t* test. Two-way ANOVA with Bonferroni correction was used to assess comparisons between multiple groups. The treatments were considered significant if the *P* < 0.05. All analysis was carried out using GraphPad Prism, version 6.0, for Windows.

## Additional Information

**How to cite this article**: Evani, S. J. and Ramasubramanian, A. K. Biophysical regulation of *Chlamydia pneumoniae*-infected monocyte recruitment to atherosclerotic foci. *Sci. Rep.* **6**, 19058; doi: 10.1038/srep19058 (2016).

## Supplementary Material

Supplementary Information
