# Supplementary material for: Biophysical regulation of Chlamydia pneumoniae-infected monocyte recruitment to atherosclerotic foci
Source: Sci Rep. 2016 Jan 20;6:19058. doi: 10.1038/srep19058 (PMC4726309; doi:10.1038/srep19058)
Supplement: Supplementary Information [file srep19058-s1.pdf]

Biophysical regulation of *Chlamydia pneumoniae*-infected monocyte recruitment to atherosclerotic foci

Shankar J. Evani, Anand K. Ramasubramanian

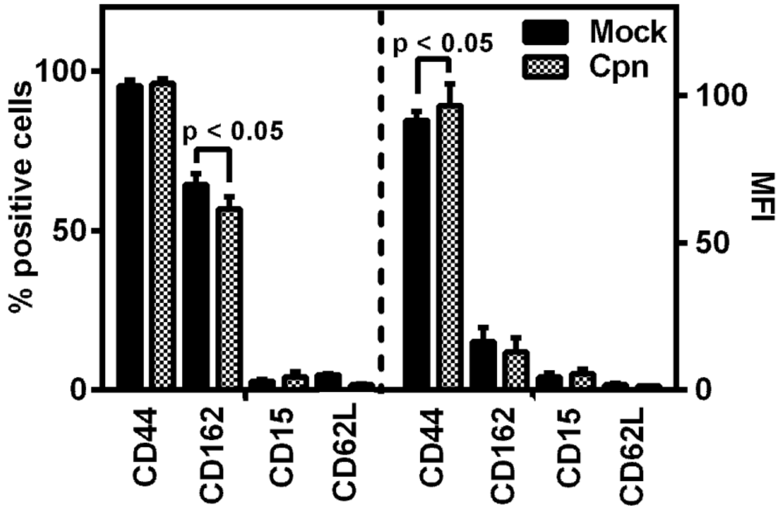

Figure S1. ***Expression levels of rolling receptors on monocytes.*** Uninfected or infected monocytes were stained for CD44/CD15/CD162/CD62L. The cells were analyzed by image stream flow cytometry for percentage cells bound with antibody against CD44/CD15/CD162/CD62L and their mean fluorescence intensity. The results are mean ± SEM of three different experiments performed in triplicate. The * denote statistically significant change in the parameters between the groups ($P < 0.05$, ANOVA).

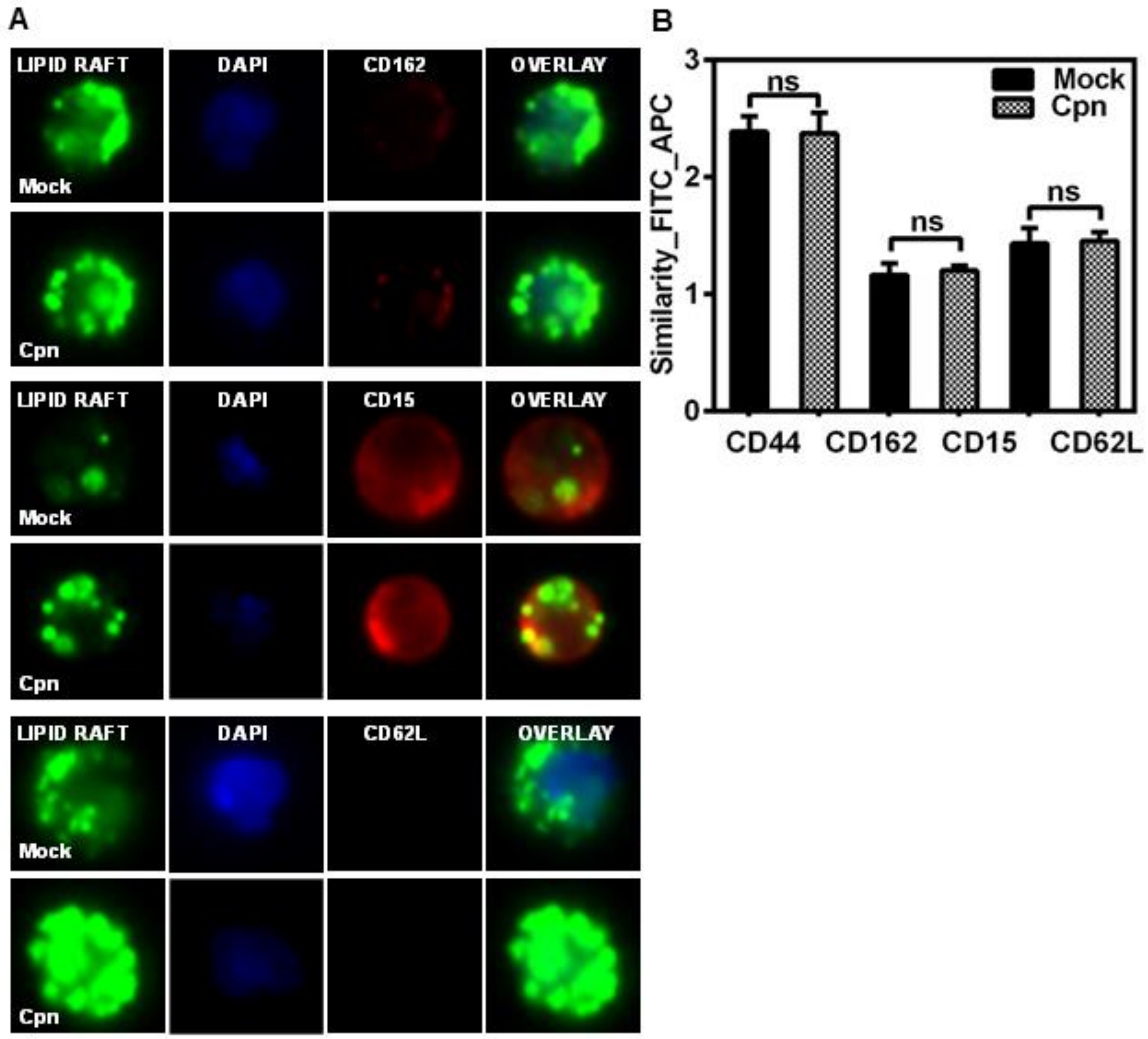

Figure S2. ***Expression levels of rolling receptors on monocytes.*** Uninfected or infected monocytes were stained for CD44/CD15/CD162/CD62L (red), lipid raft stain (green) and nucleus (Blue). The cells were analyzed by image stream flow cytometry with representative images **(A)**; and Co-localization of CD15/CD162/CD62L with lipid rafts **(B)**. The results are mean ± SEM of three different experiments performed in triplicate. The * denote statistically significant change in the parameters between the groups ($P < 0.05$, ANOVA).

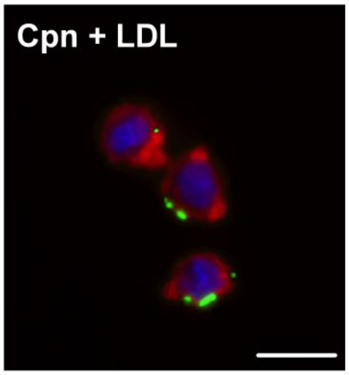

Figure S3. ***Chlamydial infection in monocytes with LDL.*** Monocytes were infected with Chlamydial EB (MOI 1) for 4 h and were exposed to 100 µg/ml of LDL for additional 4 h. The cells were stained for *C. pneumoniae* (green), actin (red), and nucleus (blue).The bar represents 20 µm. A representative image is shown and the experiment is performed three times in triplicate.

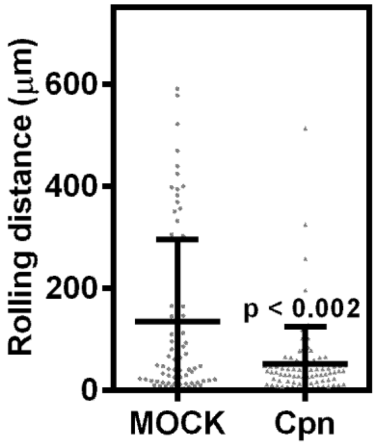

Figure S4. ***40 h infection shows increased rolling of monocytes on endothelium.*** Monocytes were infected with Chlamydial EB (MOI 1) for 40 h hours. The cells were then re-suspended at a concentration of 0.5 million/ml in media and perfused on activated aortic endothelium at 1 dyn/cm^2, and the interactions were visualized by video microscopy. Rolling distance of monocytes before firm arrest was estimated and the results are mean ± SEM of one representative experiment performed in triplicate, and the experiments were repeated three times.
